# Complement system dysfunction in autism spectrum disorder: evidence for altered C1q and C3 levels (complement system dysfunction in ASD)

**DOI:** 10.1017/neu.2025.10017

**Published:** 2025-05-22

**Authors:** Meltem Gunaydin, Ozlem Dogan, Fatih Gunay, Merve Cikili-Uytun, Özge Celik-Buyukceran, Didem Behice Oztop

**Affiliations:** 1 Department of Child and Adolescent Psychiatry, Sanliurfa City Hospital, Sanliurfa, Turkey; 2 Department of Biochemistry, Ankara University, Ankara, Turkey; 3 Department of Pediatrics, Ankara University, Ankara, Turkey; 4 Department of Child and Adolescent Psychiatry, Ankara University, Ankara, Turkey; 5 Department of Child and Adolescent Psychiatry, Igdir Public Hospital, Igdir, Turkey; 6 Ankara University Autism Research and Intervention Center, Ankara, Turkey

**Keywords:** Autism spectrum disorder, complement system, C1q, C2, C3, neuroimmune dysregulation

## Abstract

Autism spectrum disorder (ASD) is a neurodevelopmental condition characterised by impairments in social communication, repetitive behaviours, and restricted interests. Emerging evidence suggests that immune system dysregulation, particularly alterations in the complement system, may contribute to ASD pathophysiology. This study aimed to compare the serum levels of complement proteins (C1q, C2, C3, C4, MBL, L-ficolin, and hsCRP) between children with ASD and non-ASD controls. A total of 88 children (44 with ASD and 44 age- and sex-matched healthy controls) participated in this study. Complement protein levels were measured using enzyme-linked immunosorbent assay (from serum samples. The severity of ASD symptoms was assessed using standardised diagnostic tools, including the Childhood Autism Rating Scale, the Autism Behaviour Checklist, and the Repetitive Behaviour Scale-Revised. Serum C1q levels were significantly lower in the ASD group (*p* < 0.001). C3 levels were lower (*p* = 0.033), while C2 levels were slightly higher (*p* = 0.015) in the ASD group. There are no significant differences in C4, MBL, or L-ficolin levels. Logistic regression analysis identified reduced C1q levels as a significant predictor of ASD (*p* = 0.001). However, this study found no significant correlations between complement levels and ASD symptom severity scores. The findings suggest that alterations in complement system proteins, particularly reduced serum C1q levels, may be associated with ASD. Given C1q’s critical role in synaptic pruning and neuroimmune regulation, these results support the hypothesis that complement system dysfunction may contribute to the pathophysiology of ASD.

## Graphical abstract

**Figure:**
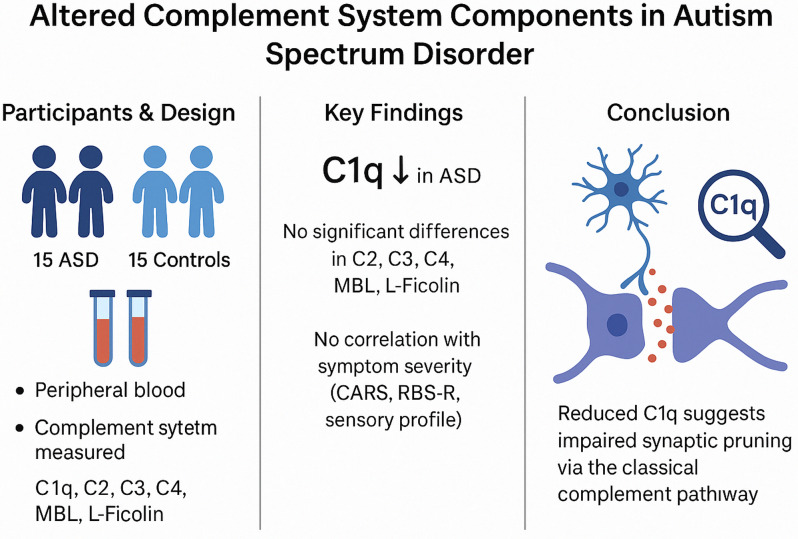

Significant outcomes

- Serum C1q levels were significantly lower in children with autism spectrum disorder (ASD) compared to healthy controls, suggesting a potential role of complement system dysfunction in ASD pathophysiology.
- Logistic regression analysis identified reduced C1q levels as a significant predictor of ASD, highlighting its potential as a biomarker for early detection.
- No significant correlations were found between complement protein levels and ASD symptom severity, indicating that while complement dysregulation may be involved in ASD aetiology, it may not directly influence symptom severity.

Limitations

- The cross-sectional design limits causal inferences about the relationship between complement system dysfunction and autism spectrum disorder development.
- The study was conducted at a single centre without randomised sampling, potentially affecting the generalisability of the findings.
- Complement protein levels were only assessed in serum samples, which may not fully reflect neuroimmune.

## Introduction

Autism spectrum disorder (ASD) is a neurodevelopmental disorder that begins in early childhood and is characterised by difficulties in social relationships and communication, stereotyped and repetitive behaviours, and restricted interests, with lifelong effects (American Psychiatric Association, 2022). According to the latest data from the Centers for Disease Control and Prevention in 2023, the prevalence of autism has risen to 1 in 36 children (Maenner, 2023). Early diagnosis of ASD is crucial for the timely implementation of behavioural interventions. Consequently, interest in ASD biomarkers has been increasing to facilitate the early identification of at-risk children (Klin, 2018).

The exact cause of ASD remains unclear; however, genetic, neuroanatomical, neurophysiological, neurochemical, neuroimmunological, and environmental factors are considered to play a role (Emberti Gialloreti & Curatolo, 2018). Various studies have demonstrated altered inflammatory responses and neuroimmune system anomalies in individuals with ASD, suggesting that immune dysregulation may contribute to its aetiology (Eissa *et al.*, 2020). One study reported that infections during pregnancy increase the risk of ASD through maternal immune activation (Jiang *et al.*, 2016; Krakowiak *et al.*, 2017). Another study found that cytokine profiles in children with ASD were abnormal (Goines & Van de Water, 2010; Ricci *et al.*, 2013), while a separate study reported an increased prevalence of autoimmune diseases in individuals with ASD (Zerbo *et al.*, 2015). These findings suggest that immune system dysfunction may actively and persistently contribute to the etiopathogenesis of ASD (Croonenberghs *et al.*, 2002).

The complement system, a component of the innate immune system, plays essential roles in immune defence, bridging innate and adaptive immunity, and clearing immune complexes and inflammatory debris. Beyond its immunological functions, it is also involved in key neurodevelopmental processes such as neurogenesis, neuronal migration, synaptic pruning, and maintaining brain homeostasis (Schafer *et al.*, 2012; Gorelik *et al.*, 2017a; Gorelik *et al.*, 2017b). It is activated through three main pathways: the classical, lectin, and alternative pathways, involving more than 30 complement proteins (Walport, 2001). C1q initiates the classical pathway by binding to antigen-antibody complexes, while MBL and L-ficolin activate the lectin pathway through recognition of microbial patterns. C2 and C4 function in both the classical and lectin pathways, and C3 is central to all pathways (Janeway *et al.*, 2001; Owen *et al.*, 2013).

The complement system also plays a crucial role in neurodevelopment, particularly in synaptic pruning. Studies in mouse models have shown that C1q, C3, and C4 contribute to the elimination of less active synapses, helping to refine neural circuits and establish mature connectivity in the central nervous system (CNS) (Lui *et al.*, 2016) (Presumey *et al.*, 2017). Similarly, studies have shown that mice deficient in C1q and C3 exhibit defects in synaptic pruning (Stevens *et al.*, 2007), and similar abnormalities have also been observed in C4-deficient mice (Sekar *et al.*, 2016). These findings suggest that dysregulation of complement activity may impact neurodevelopmental processes and contribute to disorders characterised by abnormal synaptic architecture. Alzheimer’s, Parkinson’s, and Huntington’s disease (Negro-Demontel *et al.*, 2024; Nimmo *et al.*, 2024).

There are also several studies in the literature that have specifically investigated the relationship between ASD and complement system dysfunction. Vargas *et al*. (2005) reported chronic neuroglial activation in ASD brains, suggesting involvement of complement activation.(Vargas *et al.*, 2005). Similarly, Fagan *et al*. (2017) observed increased C2, C5, and MASP1 mRNA and reduced C1q, C3, and C4 expression in the prefrontal cortex (Fagan *et al.*, 2017). These alterations parallel findings in C1q-, C3-, and C4-deficient mouse models showing increased excitatory synapses and impaired synaptic pruning (Perez-Alcazar *et al.*, 2014; Tang *et al.*, 2014). Together, these findings suggest that impaired complement-mediated synaptic pruning may contribute to the cortical hyperconnectivity and behavioural phenotypes characteristic of ASD (Magdalon *et al.*, 2020). In contrast, Mou *et al*. (2022) found elevated C4 expression in the subventricular zone, potentially reflecting neuroinflammation (Mou *et al.*, 2022). Supporting reduced C4 function, Mansur *et al*. (2021) demonstrated decreased C4 mRNA and protein in ASD astrocytes, with unchanged C5a levels (Mansur *et al.*, 2021). Additionally, genetic studies have identified a higher frequency of the C4B gene null allele in individuals with ASD (Warren *et al.*, 1991; Odell *et al.*, 2005; Mostafa & Shehab, 2010), with significantly lower plasma C4B levels also reported (Warren *et al.*, 1994).

While these studies provide insight into central complement activity, other findings point toward peripheral dysregulation as well. Corbett *et al*. (2007b) reported elevated plasma C1q in children with ASD, while Momeni *et al*. (2012) observed increased C3 fragments and Factor I activity, potentially impairing C3b-dependent phagocytosis (Corbett *et al.*, 2007b; Momeni *et al.*, 2012; Momeni *et al.*, 2012a, b). Shen *et al*. (2018) also identified increased plasma levels of C1q, C3, and C5 (Shen *et al.*, 2018). Recent proteomic analyses utilising machine learning have strengthened these findings: Shen *et al*. (2022) showed upregulation of C1q subunits (C1QA, C1QB), Zhang *et al*. (2023) found increased C2 and C3 levels, and Cao *et al*. (2023a) reported decreased C4a and increased levels of C4B-binding regulatory proteins (C4BPA, C4BPB) in ASD (Shen *et al.*, 2022; Cao *et al.*, 2023a).

Findings in the literature suggest that dysregulation of the complement system, which plays critical roles in both the immune system and synaptic pruning, may be associated with reduced synaptic pruning and altered immune responses in individuals with ASD, potentially contributing to the pathogenesis of ASD. In light of these findings, our study aims to investigate six complement system proteins (C1q, C2, C3, C4, MBL, and L-ficolin) as potential biomarkers for ASD and to assess high-sensitivity C-reactive protein (hsCRP) as a systemic marker of inflammation. Including hsCRP allows for a broader evaluation of immune activation and helps clarify whether complement alterations are accompanied by general inflammatory responses, thereby strengthening the interpretability of our results. This study seeks to determine whether complement proteins involved in all three complement pathways differ between preschool-aged children diagnosed with ASD and non-ASD controls. While previous studies have explored individual components, this study provides a comprehensive assessment of all three complement pathways in individuals with ASD. This study measured serum levels of MBL and L-ficolin and examined the associations of C1q, MBL, and L-ficolin with autism symptom domains and severity using objective assessment scales. This research has been designed based on the hypothesis that the serum levels of these complement proteins may be associated with ASD and its severity.

## Methods

### Participants

The study group consisted of 44 children (36 boys and 8 girls) aged 2–6 years who were newly diagnosed with ASD according to DSM-5 diagnostic criteria. These children were recruited between February 2022 and May 2022 from the outpatient clinic of the Department of Child and Adolescent Psychiatry at Ankara University Faculty of Medicine and the Ankara University Autism Application and Research Center. The control group included 44 age- and sex-matched children (36 boys and 8 girls) aged 2–6 years who were admitted to our child and adolescent psychiatry outpatient clinic during the same period. These children had no psychiatric diagnosis according to DSM-5 criteria and did not exhibit any cognitive delays.

For the study group, exclusion criteria included having a chronic physical, neurological, metabolic, or immunological disease and/or a genetic disorder; receiving medication for any chronic medical, psychiatric, or neurological condition within the past 2 years; and having an active infection in the last 30 days. In the control group, children who were admitted to the Department of Child and Adolescent Psychiatry outpatient clinic and the Infant Mental Health Unit for consultation services and who had no neurodevelopmental disorders, including ASD, in themselves or their families, were included. Other exclusion criteria were the same as those applied to the study group. The study was approved by the Ankara University Faculty of Medicine Non-Interventional Clinical Research Ethics Committee on 13 January 2022 (decision no.: I01-11-22).

### Diagnostic and symptom assessment

Children who met the specified inclusion and exclusion criteria and whose parents provided informed consent for participation were evaluated by a researcher specialising in child psychiatry through a diagnostic psychiatric interview based on DSM-5. All children included in both groups underwent a standardised developmental or intelligence assessment test appropriate for their developmental stage, administered in our clinic (Ankara Development Inventory or Stanford Binet Intelligence Test). Following these assessments, the children were taken to the infant observation room at the Infant Mental Health Unit, where a multidisciplinary team consisting of experienced child psychiatry faculty members, child psychologists, child psychiatry nurses, rotating paediatric residents, and psychology doctoral students conducted diagnostic confirmation and further evaluation using Crowell observation. Based on clinical observations, psychometric assessments, and information obtained from parents, the Childhood Autism Rating Scale (CARS) was completed by the researcher. Subsequently, parents of children in the ASD group were interviewed to complete the sociodemographic and clinical data form. Additionally, parents completed the Autism Behaviour Checklist (ABC) and the Repetitive Behaviour Scale-Revised (RBS-R). Parents of children in the control group were also interviewed to complete the sociodemographic and clinical data form.

The form completed by the researcher during the interview with the parents included information on sociodemographic characteristics, the mother’s pregnancy history, the child’s birth history, developmental milestones, nutritional habits, infection history, dietary habits, and the history of ASD diagnosis.

The Crowell method is a structured parent–infant play interaction used for the observational assessment of parent–child interaction. It was developed by Crowell and Feldman in 1998 (Crowell & Feldman, 1988).

CARS is an objective and measurable assessment tool that evaluates the severity of autism through the direct observation of autistic behaviours using 15 sub-items (Schopler *et al.*, 1980). The Turkish validity and reliability analysis was conducted by Gassaloğlu, Baykara, and colleagues in 2016 (Gassaloğlu *et al.*, 2016).

ABC is a 57-item assessment tool consisting of five subscales used to determine the level of autistic symptoms in an individual (Krug *et al.*, 1980). The validity and reliability study of the Turkish version was conducted by Yilmaz-Irmak et al. (Irmak *et al.*, 2007).

RBS-R is an assessment tool consisting of six subscales and a total of 43 items, used to evaluate repetitive behaviours and their severity (Bodfish *et al.*, 2000). The validity and reliability study of the Turkish version was conducted by Akçamuş et al. in 2019 (Ökcün Akcamus *et al.*, 2019).

### Blood samples

Venous blood samples were collected from participants in both groups who were deemed eligible for the study. The samples were drawn by the clinic’s nurses from the antecubital vein into biochemistry tubes at a volume of 10 mL, regardless of fasting status, between 09:00 and 10:00 a.m. The samples were transferred to the biochemistry laboratory under appropriate transport conditions within one hour and were centrifuged upon arrival. Until biochemical analysis, the samples were stored in Eppendorf tubes at −80°C. The samples were prepared according to the dilution ratios recommended by the kits, and all reagents were brought to room temperature before analysis. The determination of C1q, C2, C3, C4, C5, MBL, Ficolin-2, and hsCRP was conducted using enzyme-linked immunosorbent assay (ELISA) kits (ELK 1075, 2386, 1059, 1653, 8534, 1098, 2672, and 8534 Biotechnology ELISA, China), following the instructions provided in the kit manuals. Measurements were performed by incubating the samples on microplates coated with anti-human antibodies, followed by the application of biotin-labelled secondary antibodies and streptavidin-HRP. The reaction was developed by adding the TMB substrate solution, and the process was terminated using a stop solution. Absorbance values were measured using a microplate reader at a wavelength of 450 nm, and the results were quantitatively calculated using a standard curve. Measurement sensitivities were maintained within the ranges specified in the kit manuals, and all analyses were repeated to ensure an intra-assay coefficient of variation below 8%.

### Statistical analyses

Sample size calculations for the study parameters were performed using G*Power version 3.1.9.2. Power analyses were conducted for independent two-group *t*-tests. To detect a 5 µg/ml difference in Ficolin levels, assuming a standard deviation of 5 and aiming for a 95% power, a minimum of 23 participants per group was required (Boyajyan *et al.*, 2013). For C3 levels, detecting a 10 ng/ml difference with 80% power required at least 40 participants per group (Shen *et al.*, 2018). For C1q levels, a 6 ng/ml difference could be detected with 95% power using 16 participants per group (Corbett *et al.*, 2007a). For hsCRP, detecting a 500 ng/ml difference with 90% power required 39 participants per group (Khakzad *et al.*, 2012). Considering the statistical requirements for all parameters and aiming to meet the most conservative estimates, we planned a total of 88 participants (44 children with ASD and 44 healthy controls), which ensures adequate power across all primary outcome measures.

The normality of data distribution was assessed using the Shapiro–Wilk test. Differences in continuous variables between groups were analysed using Student’s *t*-test, while categorical variables were compared using the chi-square test. To evaluate differences in blood component levels between groups, ANCOVA adjusted for age, sex, and hsCRP levels was used. Partial correlation analysis, adjusted for age, sex, and hsCRP levels, was performed to examine the relationships between ASD and blood component levels. Additionally, multivariate logistic regression analysis was conducted to determine potential predictors of ASD. All analyses were conducted using SPSS 25.0 (IBM Corp., Armonk, NY, USA), with a significance level set at *p* < 0.05.

## Results

The participants were divided into two groups: non-ASD controls (*n* = 44) and those with ASD (*n* = 44). Table 1 presents the sociodemographic and clinical characteristics of these groups. The mean age of the non-ASD group was 43.95 ± 15.07 months, slightly higher than the ASD group’s mean age of 41.61 ± 14.10 months, but this difference was not statistically significant (*p* = .454). Both groups had identical sex distributions, with 81.8% male and 18.2% female participants (*p* = 1).

**Table 1.** Sociodemographic and clinical characteristics of participants

Variables	Healthy control (*n* = 44)	Autism spectrum disorder (*n* = 44)	
	**Mean ± SD, *n* (%)**	**Mean ± SD, *n* (%)**	***p***
**Age (month)**	43.95 ± 15.07	41.61 ± 14.10	0.454^a^
**Sex**			1.0^b^
Male	36 (81.8)	36 (81.8)	
Female	8 (18.2)	8 (18.2)	
**Family group**			0.420 ^b^
Nuclear family	35 (79.5)	40 (90.9)	
Extended family	5 (11.4)	2 (4.5)	
Single-parent (mother)	3 (6.8)	2 (4.5)	
Single-parent (father)	1 (2.3)	0 (0.0)	
**Mother**			
Maternal age (years)	34.75 ± 4.42	33.56 ± 5.75	0.283 ^a^
Education level (illiteracy)	0 (0.0)	4 (4.5)	.**041** ^**b**^
Employment (no/irregular)	19 (43.2)	30 (68.2)	0.096 ^b^
Medical history (yes)	8 (18.2)	12 (27.3)	0.309 ^b^
History of mental disorders (yes)	3 (6.89	4 (9.1)	0.694 ^b^
Psychotropic medication (yes)	6 (13.6)	8 (18.2)	0.560 ^b^
**Father**			
Paternal age (years)	37.40 ± 6.98	37.02 ± 6.88	0.795 ^a^
Education level (illiteracy)	0 (0.0)	9 (20.5)	**0.002** ^**b**^
Employment (no/irregular)	2 (4.5)	4 (9.1)	0.424 ^b^
Medical history (yes)	13 (29.5)	7 (15.9)	0.127 ^b^
History of mental disorders (yes)	0 (0.0)	3 (6.8)	0.078 ^b^
Psychotropic medication (yes)	8 (18.2)	5 (11.4)	0.367 ^b^
**Brother or sister (yes)**	9( 20.5)	15 (34.1)	0.151 ^b^
**History of mental disorders (yes)**	2 (4.5)	10 (29.4)	**0.003** ^**b**^
Cognitive developmental delay	0 (0.0)	1 (2.3)	
Attention deficit hyperactivity disorder	2 (4.5)	0 (0.0)	
Catatonia	0 (0.0)	9 (20.4)	
**Gravidity**	1.86 ± 0.73	1.69 ± 1.02	0.368 ^b^
**Abortion stories (yes)**	2 (4.5)	1 (2.3)	0.557 ^b^
**Miscarriage (yes)**	2 (4.5)	5 (11.4)	0.237 ^b^
**Unintended pregnancy (yes)**	0 (0.0)	4 (9.1)	0.116 ^b^
**During pregnancy**			
Follow-up (yes)	44 (100.0)	39 (88.6)	0.055 ^b^
High-risk pregnancy (yes)	3 (6.8)	10 (22.7)	**0.035** ^**b**^
Inpatient treatment (yes)	1 (2.3)	6 (14.0)	0.058 ^b^
Infectious diseases (yes)	0 (0.0)	6 (14.0)	**0.012** ^**b**^
Mental disorders (yes)	0 (0.0)	1 (2.3)	1.00 ^b^
Medical history (yes)	1 (2.3)	11 (25.6)	**0.002** ^**b**^
Drug use	3 (6.8)	17 (39.5)	**0.000** ^**b**^
Folic acid use (yes)	36 (81.8)	40 (90.9)	0.214 ^b^
Progesterone use (yes)	3 (6.8)	5 (11.4)	0.458 ^b^
Anticoagulant medicine use (yes)	4 (9.1)	4 (9.1)	1.00 ^b^
Psychological stress (yes)	10 (22.7)	19 (43.2)	**0.041** ^**b**^
**Preterm labour (yes)**	2 (4.5)	12 (27.3)	**0.004** ^**b**^
**Birth weight**	2.00 ± 0.00	1.81 ± 0.39	**0.003** ^**a**^
**Caesarean section (yes)**	21 (47.7)	29 (65.9)	0.085 ^b^
**Difficult birth (yes)**	1 (3.7)	5 (11.4)	0.260 ^b^
**Complicated births (yes)**	5 (11.4)	13 (29.5)	**0.034** ^**b**^
Incubator	0 (0.0)^a^	7 (15.9)^b^	**0.000** ^**b**^
Meconium aspiration syndrome	0 (0.0)^a^	1 (2.3)^a^	
Newborn jaundice	0 (0.0)^a^	5 (11.4)^b^	
**Developmental delay (yes)**	31 (70.5)		
**Age of show symptoms of autism (years)**	2.13 ± 0.64		

Note: Comparison of demographic, familial, perinatal, and early developmental variables between children with autism spectrum disorder and healthy controls.

Statistical tests used: ^a^ = Independent samples *t*-test, ^b^ = chi-square test. *p* < 0.05 was considered statistically significant (bolded).

Family structure analysis showed that 79.5% of the non-ASD control group came from nuclear families, compared to 90.9% in the ASD group, though this difference was not statistically significant (*p* = .420). Significant differences were observed in maternal and paternal education, with 4.5% of mothers and 20.5% of fathers in the ASD group being illiterate compared to none in the non-ASD control group (*p* = 0.041, *p* = 0.002, respectively). Additionally, mothers in the ASD group were less likely to be employed irregularly (43.2% vs. 68.2%, *p* = .096) and more likely to have a history of mental disorders (27.3% vs. 18.2%, *p* = .309). However, these differences were not statistically significant. The ASD group also had a higher incidence of siblings with a history of mental disorders (34.1% vs. 20.5%, *p* = .151).

During pregnancy, mothers in the ASD group reported higher rates of high-risk pregnancies (22.7% vs. 6.8%, *p* = .035), infectious diseases (14% vs. 0%, *p* = .012), and drug use (39.5% vs. 6.8%, *p* < .001). The ASD group also reported more complicated births (29.5% vs. 11.4%, *p* = .034), and newborns in the ASD group were more likely to require incubator care (15.9% vs. 0%, *p* < .001). Birth weights were significantly lower in the ASD group (1.81 ± 0.39 kg vs. 2.00 ± 0.00 kg, *p* = .003).

Table 2 compares the complement blood levels between the non-ASD group and the ASD group, adjusting for covariates (age, sex, and hsCRP). C1q levels were significantly lower in the ASD group (5.64 ± 3.81) compared to the non-ASD control group (10.95 ± 7.10), with an *F*-value of 5.384 and a *p*-value of < .001. Similarly, C3 levels were lower in the ASD group (302.53 ± 98.59) compared to the non-ASD control group (341.30 ± 107.53, *p* = .033). In contrast, C2 levels were higher in the ASD group (9.42 ± 14.41 vs. 7.02 ± 11.90, *p* = .015). Other complement components such as ficolin and MBL did not show significant differences between the two groups (*p* > .05). hsCRP levels did not significantly differ between the groups (*p* = .712).

**Table 2.** Comparison of complement protein and inflammatory marker levels between autism spectrum disorder (ASD) and non-ASD control groups

	ASD (*n* = 44)	Control (*n* = 44)			
**Complement blood levels**	**Mean ± SD**	**Mean ± SD**	***F***	***Adjusted*** ***R squared***	***p***
**C1q (ng/mL)** ^**†**^	5.64 ± 3.81	10.95 ± 7.10	5.384	0.168	< 0.001
**C3 (ng/mL)** ^**†**^	302.53 ± 98.59	341.30 ± 107.53	2.750	0.074	0.033
**C2 (ng/mL)** ^**†**^	9.42 ± 14.41	7.02 ± 11.90	3.284	0.095	0.015
**C4 (ng/mL)** ^**†**^	185.27 ± 102.14	208.63 ± 129.45	1.738	.0.33	0.149
**Ficolin (ng/mL)** ^**†**^	16.08 ± 30.46	20.42 ± 39.17	0.584	0.019	0.675
**Mannose-binding lectin (ng/mL)** ^**†**^	28.85 ± 18.57	24.85 ± 18.40	1.405	0.018	0.240
hsCRP/ng/mL) *	106.25 ± 192	90.94 ± 194.98	0.414	————	0.712
**Autism Behaviour Checklist**	43 ± 24.15	———			
**Childhood Autism Rating Scale**	40.14 ± 5.50	———			
**Repetitive Behaviours Scale – Revised**	19.65 ± 16.60	———			

Note: Comparison of serum levels of complement system components and hsCRP between autism spectrum disorder and control groups. Statistical tests used: † Analysis of covariance, adjusted for age, sex, and high-sensitivity C-reactive protein (hsCRP). * Independent samples *t*-test. Adjusted *R*^2^ and Cohen’s *d* values are provided for effect size estimation.

The correlation analysis presented in Table 3 showed no statistically significant associations between ASD severity, as measured by the ABC, CARS, and Repetitive Behaviours Scale-Revised, and blood complement levels (*p* > .05). Although weak correlations were observed between some complement components and the severity measures, none reached statistical significance (*p* > .05) (Table 3).

**Table 3.** Correlation analysis of autism spectrum disorder severity levels and blood complement levels

Correlations							
**Control variables**	*r*	***C1q***	***C3***	***C2***	***C4***	***Fikolin***	***MBL***
High-sensitivity C-reactive protein and age and sex	**Autism Behaviour Checklist**	−.095	−.045	−.258	−.139	−.032	.022
	**Childhood Autism Rating Scale**	.070	.130	−.113	.023	−.131	.110
	**Repetitive Behaviours Scale – Revised**	.067	−.085	−.248	−.117	−.042	−.039

*r*: partial correlation coefficient. Correlation analyses were adjusted for age, sex, and high-sensitivity C-reactive protein levels using partial correlation to control for potential confounding variables. No statistically significant correlations were observed.

**Figure 1.**
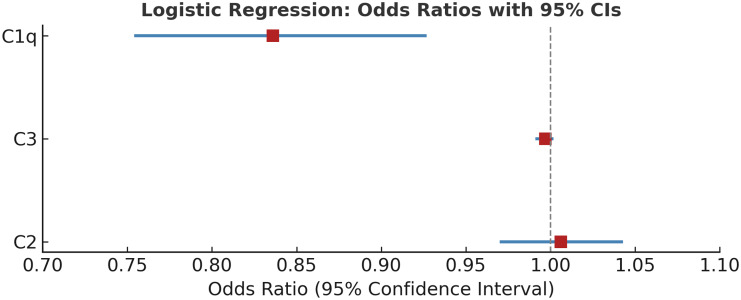
Forest plot showing odds ratios (OR) and 95% confidence intervals (CI) from multivariate logistic regression analysis assessing the association between complement proteins (C1q, C3, C2) and autism spectrum disorder. C1q showed a statistically significant association (OR = 0.836, 95% CI: 0.755–0.926, *p* < 0.01), while C3 and C2 were not significant predictors.

The multivariate logistic regression analysis (Table 4) identified C1q as a significant predictor of ASD (*B* = −0.179, *p* = .001), indicating that lower levels of C1q are associated with a higher likelihood of ASD (Exp(B) = 0.836). The model correctly classified 71.6% of cases and had a chi-square value of 20.627 and a Nagelkerke R^2^ of 0.279, suggesting a moderate explanatory power. Neither C3 nor C2 levels were significant predictors in this model (*p* > .05). This implies that although C1q may be a promising biomarker for further investigation, other complement components may not independently predict ASD (Table 4) (Fig. 1).

**Table 4.** Multivariate logistic regression analyses of potential predictors of autism spectrum disorder

					95% CI for EXP(B)	
	*B*	SE	Sig.	Exp(B)	Lower	Upper
**Constant**	2.409	0.905	**0.008**	11.121		
**C1q**	−0.179	0.052	**0.001**	0.836	0.755	0.926
**C3**	−0.003	0.002	0.151	0.997	0.992	1.001
**C2**	0.006	0.018	0.740	1.006	0.971	1.042

Note: Results from logistic regression, model summary; chi-square = 20.627, *p* < 0.001, percent correct classification 71.6% with Nagelkerke *R*^2^ of 0.279 *p* < 0.05 statistically significant (bold values).

### Discussion

In our study, peripheral parameters of the complement system, which plays an important role in the immune system and neuronal development, were evaluated in ASD and non-ASD groups. The fact that there were no differences between the groups in terms of age and gender in our study can be considered a positive result in terms of controlling confounding variables as much as possible in comparisons. Family history of psychiatric illness was found to be higher in the ASD group compared to the control group, and this finding was consistent with previous studies (Lauritsen *et al.*, 2005). In addition, it was determined that unplanned pregnancy, maternal infection during pregnancy, and stress rates were higher in the ASD group. These findings are consistent with the literature suggesting that maternal infection and stress increase the risk of ASD through epigenetic mechanisms (Kinney *et al.*, 2008; Jiang *et al.*, 2016). The higher prevalence of preterm birth, low birth weight, and birth complications in the ASD group is also in line with studies highlighting these factors as relevant risk factors (Schendel & Bhasin, 2008; Zhang *et al.*, 2019). Furthermore, when evaluating the clinical characteristics of children in the ASD group, the most frequently observed symptoms were speech delay and lack of response to name, which are widely reported in the literature (Bedford *et al.*, 2016).

Studies on brain tissues of individuals diagnosed with ASD have shown reduced synaptic pruning, increased dendritic spine density, and abnormal synaptic growth favouring excitatory synapses (Tang *et al.*, 2014). Parallel findings in C1q-deficient mouse models have shown reduced synapse elimination and increased excitatory connectivity (Fonseca *et al.*, 2004; Stevens *et al.*, 2007; Stephan *et al.*, 2013), suggesting a potential role of C1q in ASD-related neurodevelopmental processes (Fang *et al.*, 2015). In line with this hypothesis, our study found significantly lower serum C1q levels in children with ASD compared to non-ASD controls. This finding may indicate that complement system dysfunction contributes to disrupted synaptic pruning in ASD. However, results in the literature are not entirely consistent. For example, Corbett *et al*. (2007) found significantly elevated serum C1q levels in children with ASD (Corbett *et al.*, 2007b). Methodological differences may account for this discrepancy. Their study involved older children (4–6 years) diagnosed using DSM-IV and receiving medications like SSRIs or antipsychotics. In contrast, our sample consisted of medication-naïve, newly diagnosed children (aged 2–6 years) evaluated under DSM-5 criteria. These clinical factors may influence immune marker expression.

Interestingly, recent proteomic studies have reported increased expression of individual C1q subunits, specifically C1QA and C1QB (Shen *et al.*, 2022) and C1QC (Cao *et al.*, 2023a), in individuals with ASD. These subunits are essential components of the full C1q molecule. Their localised upregulation may reflect CNS-specific compensatory responses. However, our ELISA-based analysis focused on total serum C1q levels and did not assess subunit expression. This discrepancy may reflect methodological differences (proteomic vs. ELISA-based analysis) or compartmental expression patterns, where upregulation in brain tissue does not necessarily translate to elevated serum levels. Peripheral factors such as altered synthesis, degradation, or clearance of C1q may also contribute. To address these inconsistencies, future multicentre studies using integrated approaches that assess both total C1q levels and individual subunit expression in central and peripheral compartments are needed. Other complement-related proteins have also been linked to ASD. Raeisy *et al*. (2022) reported elevated serum and gene expression levels of C1q/TNF-related protein-1 (CTRP1), potentially contributing to synaptic dysfunction in ASD (Raeisy *et al.*, 2022). While our study focused solely on C1q, these results underscore the need to assess multiple complement-related proteins in future research.C1q also plays roles in other psychiatric disorders. Its levels have been inconsistently reported in schizophrenia (Idonije *et al.*, 2012; Laskaris *et al.*, 2019) but shown to be elevated in major depressive disorder and bipolar disorder, likely due to its role in neuroinflammation and microglial activation (Yao & Li, 2020; Yu *et al.*, 2021). These findings support the broader relevance of C1q in neuropsychiatric pathophysiology. Functionally, C1q is known to mediate immune regulation and synaptic pruning through microglial activation and clearance of apoptotic cells (Cho, 2019).

The reduced serum C1q levels observed in our study support the hypothesis that immune dysregulation and impaired neuroinflammatory processes may contribute to ASD pathophysiology. Given its central role in neuroimmune interactions and synaptic refinement, C1q could be further investigated as a potential biomarker for ASD. However, its diagnostic utility remains unclear, as it is not yet known whether peripheral levels of C1q reliably reflect CNS activity. It is also possible that low peripheral C1q reflects reduced central levels, potentially affecting synaptic pruning. While complement proteins are synthesised locally in both the CNS and periphery, they generally do not cross the blood-brain barrier (BBB) unless it is compromised (Van Beek *et al.*, 2003; Schafer *et al.*, 2012). Given reports of BBB disruption in ASD (Kealy *et al.*, 2020), this remains a plausible mechanism, though further research is needed to clarify the link between peripheral and central C1q in ASD.

To our knowledge, few studies have directly examined C1q levels in relation to autism severity. Our study is among the first to explore this association, but we found no significant correlation between serum C1q and symptom scores. Considering the multifactorial and heterogeneous nature of ASD, the absence of a direct relationship may reflect the complexity of the disorder and the influence of other biological or environmental factors. Nonetheless, current data are insufficient to draw firm conclusions, and further large-scale, multicentre studies are needed to better elucidate the role of C1q in ASD pathophysiology and its potential relationship to symptom severity.

Moving beyond C1q, we also assessed C3 and C4, central components of all complement pathways. In our study, serum C3 levels were found to be lower in children with ASD compared to non-ASD controls; however, this difference was not significant in logistic regression analysis. Similarly, no significant difference was observed in serum C4 levels between the groups. Consistent with our C3 findings, X. Cao et al. (2023) also reported no significant differences in serum C3 levels between ASD and control groups in a recent proteomic analysis incorporating machine learning. Although their study did not assess total C4 levels, they reported decreased levels of C4a, a degradation product of C4, and increased levels of regulatory proteins that bind C4b (C4BPA and C4BPB) in individuals with ASD. These results suggest that complement activation and regulation may be altered in ASD, even when total complement protein levels appear unchanged (Cao *et al.*, 2023a). This highlights the importance of future studies that evaluate both quantitative and functional aspects of the complement system in ASD populations.

Other studies offer mixed results. A study by Ashaat *et al*. (2017) involving children aged 6–12 years similarly reported no significant group differences in C3 and C4 levels. However, unlike our findings, they observed a significant correlation between autism severity and C4 levels (Ashaat *et al.*, 2017). Differences in methodology, including the use of ICD-10 diagnostic criteria, nephelometry-based measurements, and an older sample (6–12 years), may partly explain these discrepancies. Our sample consisted of younger, newly diagnosed children (2–6 years), assessed using DSM-5 and ELISA, with no medication exposure – reducing potential confounders. Zhang *et al*. (2023), using proteomic and machine learning methods, reported elevated C3 levels in ASD patients aged 2–3 years, suggesting peripheral activation of the classical complement pathway (Zhang *et al.*, 2023). This finding contrasts with ours and may reflect differences in age range, sample size, and analytical approach. Similarly, Al-Ameen *et al*. (2020) reported significantly elevated serum C3 and C4 levels in children with ASD aged 2–12 years (Al-Ameen *et al.*, 2020). This divergence may be attributed to several factors: their broader age range (2–12 years), smaller sample size, and reliance on DSM-IV criteria. However, their broader age range, smaller sample size, use of DSM-IV, and reliance on immunodiffusion rather than ELISA, without controlling for confounders such as infection or medication use, may explain the inconsistency when compared to our results.

Complement-related genes may also play a role. Torres *et al*. (2001) found a strong association between the C4B null allele and ASD (Torres *et al.*, 2001). Similarly, Mansur *et al*. (2021) reported reduced C4 expression in induced pluripotent stem cell-derived astrocytes from individuals with ASD, suggesting reduced complement-mediated synaptic pruning (Mansur *et al.*, 2021).

Conversely, Mou *et al*. (2022) found increased C4 expression in the subventricular zone of ASD brains, possibly reflecting age-related or region-specific effects (Mou *et al.*, 2022). Gallego *et al*. (2021) also showed CNS C4 levels increase with age – relevant since our participants were younger (2–6 years) (Gallego *et al.*, 2021). Future studies should examine C4 levels across developmental stages and in CSF versus serum.

Complement-related synaptic alterations have also been reported in both human and animal models. A 2020 review reported increased dendritic spines and glutamate synapses in ASD brains, along with similar synaptic abnormalities in C1q- and C3-deficient mice, and impaired elimination of retinogeniculate synapses in C4-deficient mice. Additionally, C3-deficient mice exhibited ASD-like behaviours, such as social deficits and repetitive movements in the prefrontal cortex. These findings suggest that impaired complement-mediated synaptic pruning may contribute to cortical hyperconnectivity and behavioural features in ASD (Magdalon *et al.*, 2020). The finding that C1q levels were significantly lower in individuals with ASD in our study supports this hypothesis. In contrast, the finding of no difference in C3 and C4 levels seems to be an inconsistent result. However, it is evident that more studies investigating the relationship of these three important proteins in the complement system with synaptic pruning in the aetiology of ASD are needed.

In our study, serum C2 levels were higher in children with ASD compared to healthy controls; however, this difference was not statistically significant in the logistic regression analysis. Similarly, a recent proteomic study by X. Cao *et al*. (2023), which used machine learning and multiple reaction monitoring techniques, also reported no significant difference in C2 levels between ASD and control groups (Cao *et al.*, 2023a). In contrast, Shen *et al*. (2022) found elevated C2 levels in individuals with ASD through proteomic analysis of plasma and peripheral blood mononuclear cells (PBMC). Their sample included participants carrying de novo mutations associated with ASD, and the authors suggested that increased C2 expression may reflect activation of the classical complement pathway in ASD (Shen *et al.*, 2022). These inconsistencies may stem from differences in analytical techniques (e.g. proteomics vs. ELISA), sample sources (plasma, serum, PBMC), and participant characteristics such as age, genetic background, and clinical phenotype. Further studies, including functional complement assays, are warranted to better understand their contribution to ASD.

Additionally, no significant differences were observed in serum MBL and L-ficolin levels between individuals with ASD and non-ASD control groups, and to our knowledge, these proteins have not been directly studied in ASD populations before. However, some evidence suggests that components of the lectin pathway may be altered in ASD. For instance, a recent proteomic analysis by Tang *et al*. (2025) identified elevated MASP-2 levels in children with ASD, indicating potential activation of the MBL-associated complement cascade (Tang *et al.*, 2025). Given that MASP-2 functions in concert with MBL to initiate the lectin pathway, future studies should consider evaluating both proteins simultaneously to better understand their joint contribution to ASD-related immune dysregulation. Moreover, studies on schizophrenia have reported similar findings. For example, MBL and MASP-2-mediated complement activation capacity has been reported to be elevated in schizophrenia patients (Mayilyan & Sim, 2020), and increased L-ficolin activity has also been noted (Gracia *et al.*, 2021). These findings suggest that haemolytic activation of MBL and L-ficolin may play a role in the aetiology of schizophrenia. However, in our study, only the serum levels of these proteins were evaluated, and their haemolytic activation was not examined, which may have limited our comparison of the similar roles of these proteins in the aetiology of schizophrenia and ASD. In conclusion, it is possible that immunologic mechanisms function differently in ASD than in schizophrenia or that these proteins do not play a significant role in the aetiology of ASD. There is a clear need for extensive and further research to better understand the relationship between these complement proteins (MBL and L-ficolin) and ASD.

While no significant correlation was shown between the serum levels of complement proteins (C1q, C2, C3, C4, MBL, and L-ficolin) and the total or subscale scores of autism severity in our study, similar null findings were also reported by X. Cao *et al*. (2023). In their proteomic analysis of ASD, no significant associations were found between C2 or C3 levels and clinical symptom severity, supporting our results (Cao *et al.*, 2023a). In contrast, prior studies in schizophrenia populations have demonstrated such associations. For instance, Laskaris *et al*. (2019) observed a negative correlation between C3 levels and both the positive and negative subscales of the PANSS, as well as a positive correlation with serum C4 levels, suggesting that C4 may be predictive of symptom severity in schizophrenia (Laskaris *et al.*, 2019). Similarly, Yin Cao *et al*. (2023) found that in patients experiencing a first episode of schizophrenia, serum C2 and C3 levels were positively correlated with the SANS and SAPS scores, respectively (Cao *et al.*, 2023b).

The absence of significant correlations between complement levels and ASD symptom severity may be explained by the clinical and biological heterogeneity of ASD, which can mask direct biomarker–symptom relationships. It is also possible that complement dysfunction plays a role in early neurodevelopment, contributing to ASD risk without directly reflecting current symptoms.

### Strengths and limitations

A major strength of the current study is its comprehensive evaluation of complement proteins representing all three pathways in ASD, which has been infrequently addressed in prior research. In addition, the fact that individuals diagnosed with ASD and non-ASD controls were matched in terms of gender, that a multidisciplinary team evaluated the entire research group, and that hsCRP was used in the comparison of inflammation levels increases the originality and methodological soundness of our study.

However, our study also has some limitations. The ADOS structured diagnostic tool could not be applied because it was not available in our clinic during this period.

Nevertheless, the multidisciplinary team evaluating the participants included a certified ADOS practitioner and had extensive experience in early childhood autism assessment. Other limitations include the cross-sectional and single-centre design, non-random sample selection, and the use of serum samples only for biochemical analyses. Regulatory proteins (e.g. Complement Factor I) and cerebrospinal fluid (CSF) markers, which may more directly reflect central immune processes, were not assessed. Moreover, the ELISA technique employed did not allow for differentiation between individual C1q subunits (C1QA, C1QB, C1QC) or between C4A and C4B isoforms, which may have limited the depth of molecular interpretation and contributed to discrepancies with prior genetic and proteomic findings. Lastly, the control group consisted of children referred to a psychiatric outpatient clinic rather than typically developing peers, which may limit the generalisability of the results to the broader paediatric population.

## Conclusion

To our knowledge, this study is among the few to evaluate MBL and L-ficolin collectively in ASD and to explore their association with symptom severity. No significant group differences were found in serum levels of C1q, C2, C3, C4, MBL, and L-ficolin, suggesting a limited role in ASD pathophysiology. In contrast, reduced C1q levels may point to impaired synaptic pruning and altered neuroimmune signalling in ASD. Given its functional role, C1q could be further explored as a potential biomarker, though its diagnostic relevance requires additional validation. Future multicentre studies integrating both serum and CSF analyses, as well as genetic and proteomic profiling, are needed to confirm these findings.
